# Addressing the Shortage of Health Professionals in Official Language Minority Communities to Strengthen Retention Strategies for the Benefit of New Brunswick Francophone and Acadian Communities: Protocol for a Mixed Methods Design

**DOI:** 10.2196/41485

**Published:** 2023-05-03

**Authors:** Stéphanie Collin, Claire Johnson, Anik Dubé, Marie-Eve Laforest, Martin Lauzier, Michel H Landry, Manon Cormier, Brigitte Sonier-Ferguson

**Affiliations:** 1 École des hautes études publiques, Secteur administration publique et gestion des services de santé Faculté des arts et des sciences sociales Université de Moncton Moncton, NB Canada; 2 Département de relations industrielles Université du Québec en Outaouais Gatineau, QC Canada; 3 Centre de formation médicale du Nouveau-Brunswick Université de Moncton Moncton, NB Canada; 4 Réseau de santé Vitalité Bathurst, NB Canada

**Keywords:** human resource shortage, retention, health care professional, health care provider, shortage, health care professionals’ retention, registered nurse retention, physicians’ retention, retention factors, Francophone communities, linguistic minorities, rural communities, minority, minorities, language, rural, ethnic, French, Francophone, Acadian, Canada

## Abstract

**Background:**

COVID-19 has highlighted already existing human resource gaps in health care systems. New Brunswick health care services are significantly weakened by a shortage of nurses and physicians, affecting regions where Official Language Minority Communities (OLMCs) reside. Since 2008, Vitalité Health Network (the “Network”), whose work language is French (with services delivered in both official languages, English and French), has provided health care to OLMCs in New Brunswick. The Network currently needs to fill hundreds of vacant physician and nurse positions. It is imperative to strengthen the network’s retention strategies to ensure its viability and maintain adequate health care services for OLMCs. The study is a collaborative effort between the Network (our partner) and the research team to identify and implement organizational and structural strategies to upscale retention.

**Objective:**

The aim of this study is to support one of New Brunswick health networks in identifying and implementing strategies to promote physician and registered nurse retention. More precisely, it wishes to make 4 important contributions to identify (and enhance our understanding of) the factors related to the retention of physicians and nurses within the Network; determine, based on the “Magnet Hospital” model and the “Making it Work” framework, on which aspects of the Network’s environment (internal or external) it should focus for its retention strategy; define clear and actionable practices to help the Network replenish its strength and vitality; and improve the quality of health care services to OLMCs.

**Methods:**

The sequential methodology combines quantitative and qualitative approaches based on a mixed methods design. For the quantitative part, data collected through the years by the Network will be used to take stock of vacant positions and examine turnover rates. These data will also help determine which areas have the most critical challenges and which ones have more successful approaches regarding retention. Recruitment will be made in those areas for the qualitative part of the study to conduct interviews and focus groups with different respondents, either currently employed or who have left it in the last 5 years.

**Results:**

This study was funded in February 2022. Active enrollment and data collection started in the spring of 2022. A total of 56 semistructured interviews were conducted with physicians and nurses. As of manuscript submission, qualitative data analysis is in progress and quantitative data collection is intended to end by February 2023. Summer and fall 2023 is the anticipated period to disseminate the results.

**Conclusions:**

Applying the “Magnet Hospital” model and the “Making it Work” framework outside urban settings will offer a novel outlook to the knowledge of professional resource shortages within OLMCs. Furthermore, this study will generate recommendations that could contribute to a more robust retention plan for physicians and registered nurses.

**International Registered Report Identifier (IRRID):**

DERR1-10.2196/41485

## Introduction

### Background

In Canada and elsewhere, COVID-19 has highlighted the human resource gaps already present in health care systems. In New Brunswick, health care services are weakened by a shortage of registered nurses and physicians, mainly affecting rural areas, where Francophone and Acadian communities are concentrated and considered as Official Language Minority Communities (OLMCs) [1]. These OLMCs have greater social and health disparities (eg, smoking and obesity) [2] and are older than their Anglophone counterparts, which are the majority population in New Brunswick [3,4]. In other words, being part of a linguistic minority group is a potential barrier to health care and services access and makes people vulnerable to health inequities [3-5].

Since 2008, a total of 2 health networks have been providing health services and care to the population of New Brunswick: Vitalité Health Network (the “Network”) and Horizon Health Network. They are required by the Regional Health Authorities Act to provide services to users in the official language of their choice (English or French). It is worth mentioning that the same law stipulates that the language of operation of Horizon Health Network is English, whereas it is French for the Vitalité Health Network. The Network, our partner, covers 4 geographic regions, 3 of which are in rural areas where many Acadian and Francophone communities reside. The fourth zone includes a more urban area, where both health networks provide health services. The New Brunswick population currently totals 775,610 citizens, 30% of whom report French as their first language [6]. The Network manages and delivers health care and services to Francophone citizens in the province, representing approximately 241,100 people living in the 4 geographic territories [6]. It has approximately 7700 part-time and full-time employees, including 560 physicians [7].

The latest data show that there are 100 vacant physician positions [8] and 200 vacant registered nurse positions [9]. For the past year, a shortage of staff has caused the closure of the obstetric services in the Restigouche region [10]. Also, the departure of oncologists has recently affected the Dr Georges-L.-Dumont University Hospital Centre in Moncton. Several media papers have addressed these issues [11]. Provincial projections predict a critical shortage of more than 1300 registered nurses in the next 5 to 7 years, and approximately 35% of family physicians will retire [12]. Given these staff shortages, combined with the global COVID-19 pandemic, it is imperative to strengthen the Network’s retention strategies to ensure its viability and reduce the health disparities in OLMCs.

### Theoretical Frameworks

The conceptual framework is based on the “Making it Work” framework [13,14] and the “Magnet Hospital” model [15], in addition to the delivery of health care services in OLMCs. The inclusion of the linguistic minority variable will lead us back to our objectives or research questions and will provide theory-based results, increase credibility, and foster transferability to practice settings [16].

### The “Making it Work” Framework

**Figure 1:**
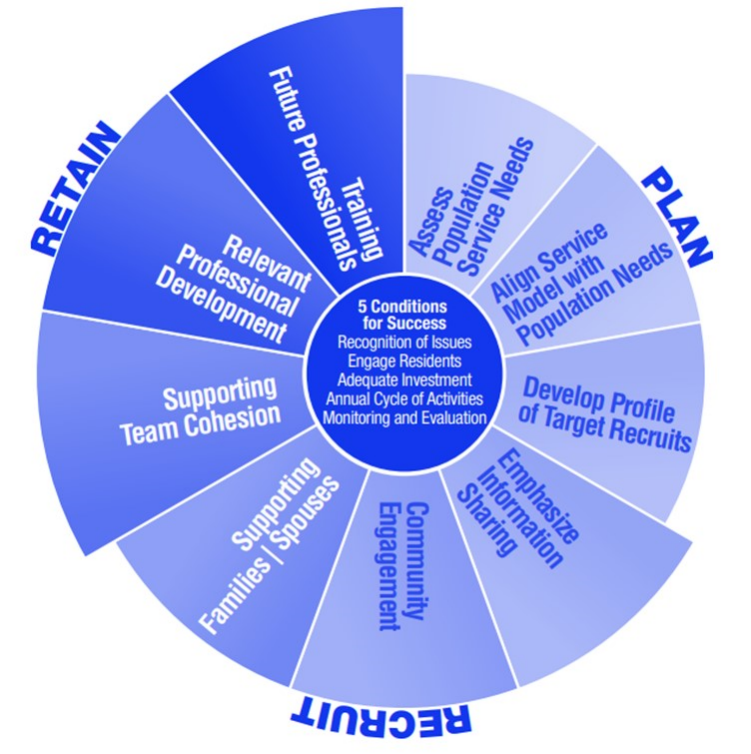
Making it Work framework (adapted from École de médecine du Nord de l’Ontario [20], which is published under Creative Commons Attribution 4.0 [CC BY 21]).

The “Making it Work” framework, which focuses on rural and remote recruitment and retention, is based on 9 key strategic elements that primarily address determinants of the environment that are grouped under 3 main activities (ie, planning, recruiting, and retaining) and are briefly described [13,17] (see Figure 1). The Network’s Board of Directors decided in 2020 to take a closer look at this framework, which has been the focus of a framework in Northern Ontario [14,18]. The framework was launched in 2011 by an international collaborative, addressing the challenges of recruiting and retaining health professionals in rural and remote northern settings and has been the focus of the Northern Ontario School of Medicine, which has sites throughout the north of Ontario, 1 being in the OLMC of Sudbury [19].

*Planning* thus involves assessing the population’s health needs and aligning service delivery models with them [17]. This task also includes developing job profiles for the professionals the organization wishes to recruit, including skills specific to working in rural and remote settings [17]. *Recruitment* focuses on sharing information about the work environment, practice, population served, and available housing in the area [17]. Recruiting also involves consideration of community engagement and support for the professional’s family, such as employment opportunities for the spouse. Finally, elements included in the *retention* component of the model are related to team cohesion, professional development, and training [17].

In total, 5 conditions are contingent on the success of the framework [17,22]: acknowledgment of the unique challenges of rural and remote areas (eg, the unique aspects of living and working in these areas compared to urban settings), engagement of communities and consideration of their perspectives (in workforce planning or policy-making), adequate investments (targeting professional development and training internships, in particular), an annual cycle of activities (as opposed to actions driven by urgency) that promote recruitment and retention (like attention-drawing job descriptions or performance standards), and finally, continuous monitoring and evaluation (to modify interventions based on experiences and improve quality to meet the needs of the population). Conjointly, these conditions promote the establishment of a stable and appropriately qualified professional workforce in rural and remote communities, in this case, the OLMCs.

### The Magnet Hospital Model

The Magnet Hospital model emerged in the early 1980s when the American Academy of Nursing appointed a task force to study the facilitating factors and barriers to professional nursing practice in hospitals [15]. McClure et al [23] revisited the work done by these forerunners, focusing on organizational culture and quality of care:

We found that all these settings had a commonality: their corporate cultures were totally supportive of nursing and of quality patient care. What we learned was that this culture permeated the entire institution. It was palpable, and it seemed to be almost a part of the bricks and mortar. Simply stated, these were good places for all employees to work (not just nurses), and these were good places for patients to receive care. The goal of quality was not only stated in the mission of these institutions, but it was lived on a daily basis.

p. 199

**Figure 2:**
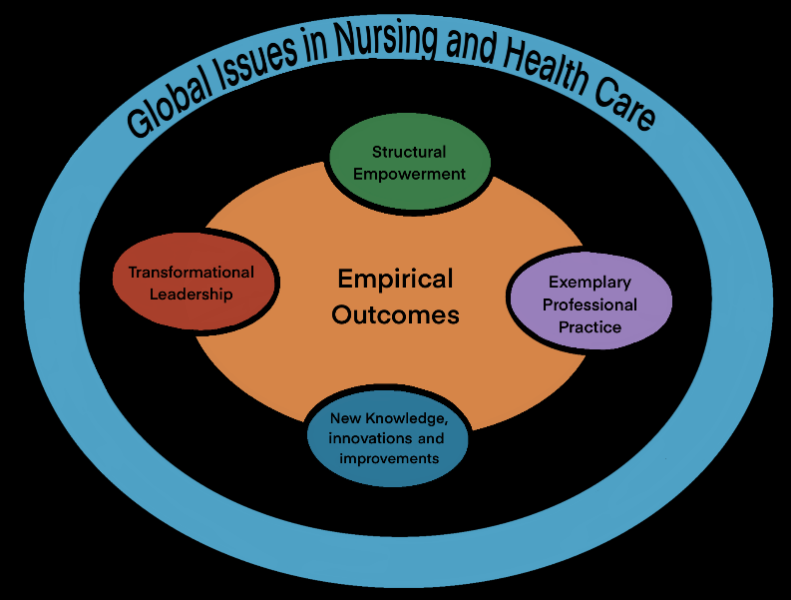
An evaluation of the nursing practice environment and successful change management using the new generation Magnet Hospital model [25].

Thus, the Magnet Hospital model, or the “magnet” hospital, means “literally, the ability to retain and attract staff, and figuratively, the ability to foster well-being to their patients” [24]. The evolution of this model over the past few decades led to the model presented in Figure 2.

The model [25] in Figure 2 builds on areas that are primarily part of the work environment: (1) transformational leadership (ie, “magnet” managers at all organizational levels), (2) exemplary professional practices (eg, the ethic of caring), (3) structural empowerment (eg, decentralized decision-making), (4) new knowledge, innovation, and improvements (eg, research- and evidence-based decision-making), and (5) empirical outcomes (eg, quality of care and organizational goals) [25,26]. These components cannot be separated from the overall context, relating to, for example, the global pandemic, and all have implications for employee retention, optimal service delivery, and quality of clinical care [27,28].

### Shortage of Health Care Professionals and Access to Health Care in OLMCs

The shortage of registered nurses and physicians is particularly acute in rural areas, where are concentrated OLMCs [1,3]. These regions have difficulty attracting and retaining professionals who generally do their training and start working in urban settings [29]. Also, a preference for acute care clinical settings in urban areas and the mobility of professionals make recruitment and retention challenges difficult to overcome by rural and remote OLMCs [30].

A study [30] has shown that one of the factors affecting the retention of professionals in OLMCs is the quality of the work environment. For example, the quality of social ties, the sense of belonging, the diversity of the environment, both ethnic and linguistic, and the working conditions influenced their desire to remain in their position [30].

For OLMCs, a shortage of registered nurses and physicians can lead to inadequate access to health services and negatively impact initiatives to meet their health needs, such as those in prevention and promotion [31,32]. The provision of quality and safe health care services to OLMCs is, in other words, linked to the availability of competent professional resources [33]. Furthermore, studies have shown that the challenges to offering services to the linguistic minority group are due to the lack of professionals who are able to provide these services within OLMCs [34,35]. The smaller pool of bilingual professionals can thus have consequences on the health of these communities [36-38].

### Conceptual Framework and Research Questions

**Figure 3:**
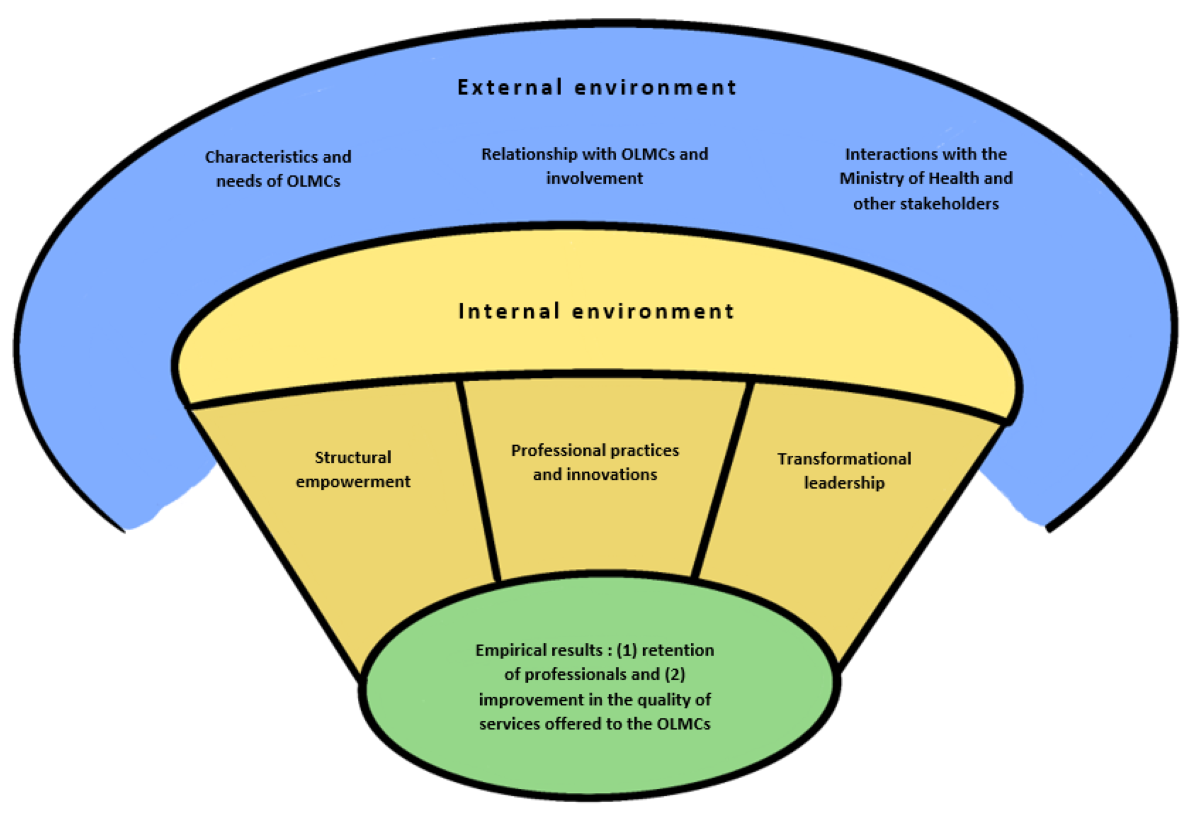
Factors influencing the retention of Registered Nurses and Physicians Working in OLMCs. OLMCs: Official Language Minority Communities.

The conceptual framework presented in Figure 3 shows that varied factors in the internal and external environment influence the retention of registered nurses and physicians. The “Magnet Hospital” model provides key work environment components for understanding the retention capacities of these health professionals that are also essential to quality patient care, whereas the “Making it Work” concept helps us analyze the retention factors through the lens of strategic elements crucial to accessing high-quality health services in rural and remote environments.

The internal environment of the health organization (in yellow in Figure 3) has three components: (1) structural empowerment, (2) professional practices and innovations, and (3) transformational leadership. *Structural empowerment* refers to a structure that is decentralized and debureaucratized [39]. This component includes recognition measures such as awards or scholarships for innovative projects [40] and employee satisfaction with salary, workload, and work-life balance. It also considers other vital elements such as competitive pay, adequate staffing, a sense of control over decision-making and practice, and perceived stress levels [15,24].

*Professional practices and innovations* are associated with a philosophy of excellence in care that emphasizes evidence-based skills and best practices, a focus on quality improvement through innovative approaches, and the value placed on professional development [15,39]. This component indicates the importance of ensuring that resources are available to new employees like mentorship, access to advice, and other resources to help with their integration. A collaborative relationship between registered nurses and physicians and autonomy based on professional standards are all part of this component [15,27]. Ethical values in caring and transparency are linked to better care for people needing health care and services while helping with their self-fulfillment [24].

*Transformational leadership* refers to close supervision that is characterized by a manager who is visionary, respectful, attentive to their collaborators, stimulating, and mobilizing [24,41]. To counteract burnout, the caring approach to management prioritizes social ties and team cohesion as well as dialogue and trust [41]. In addition, the practice of supportive management must take place in a stimulating work environment to be effective.

The external environment of the health organization (in blue in Figure 3) is made up of three main dimensions: (1) the characteristics and needs of OLMCs; (2) the relationship established between the health organization and the OLMCs, and their involvement in attracting and retaining professionals; and lastly (3) interactions with the Department of Health and other relevant actors (such as training institutions).

The *characteristics and needs of OLMCs* focus on the particularities of the populations served by the health organization. In New Brunswick, the territory served by the Network is composed primarily of Francophone and Acadian populations, many of which are in rural areas. These citizens are generally more disadvantaged in terms of socioeconomic status and are older than elsewhere in the province or the country [42]. The health needs of the OLMCs served by the Network differ from one health zone to another and even from one city to another and must be reevaluated on an ongoing basis. A service model that does not meet the needs of rural and remote communities can lead to burnout and job dissatisfaction for its employees [14].

The *involvement of OLMCs* in efforts to counter the departure of health professionals is reported in the literature on the "Making it Work" concept [13,14]. While the involvement of rural and remote communities is crucial when recruiting health care professionals [14], policies that encourage their active and ongoing participation are also essential to ensure the retention and stability of medical personnel [43].

The interactions with the *Department of Health and other relevant actors* go hand in hand with the involvement of OLMCs, as it is linked to the relational dynamics in the planning stage between the health organization and other key players, particularly the Department of Health, training institutions, and nongovernmental organizations. Decisions made by actors of the external environment, sometimes outside the control of the Network, can affect the intention to stay of professionals. Furthermore, without adequate investments, such as in professional development, efforts remain focused on solving urgent problems (vacancies) at the expense of an ongoing strategy to ensure the stability of the skilled workforce [17].

Finally, the empirical results (in green in Figure 3) are influenced by “what’s going on” in the environment (internal and external) of the health care organization. Factors emanating from these 2 spheres affect the commitment and retention of registered nurses and physicians responsible for improving the quality of services offered to OLMCs.

### Main and Specific Objectives

The overall objective of this study is to support one of New Brunswick health networks in identifying and implementing concrete strategies that take into consideration its environment to promote physician and registered nurse retention.

Two specific objectives are derived from the general research objective: (1) to identify current staffing trends and examine the turnover rate in each geographical zone of the Network to determine where the most significant challenges are with recruitment and retention; (2) to identify and understand the factors that affect the retention of physicians and registered nurses (a) at the operational level, to determine the factors that make the Network’s environment (internal and external) attractive, such as the working and living conditions of professionals, and (b) at the strategic level, to make concrete and valuable recommendations to our main partner, the Network.

## Methods

### Methods Overview

This study will use a sequential mixed methods design that combines quantitative and qualitative approaches [44]. This design offers a more comprehensive technique to better understand phenomena and nuance data interpretation [45].

Initially, secondary data will be compiled within existing sources to identify current organizational trends and inform our qualitative data collection strategy. In other words, the quantitative analysis of secondary data will guide the qualitative approaches’ recruitment process. Specifically, we will assess job vacancy and turnover rates for nurses and physicians to target the more problematic geographical zones and departments concerning retention. Turnover rates are commonly evaluated as an indicator of retention of hospital staff, and it is especially used for nurses [46-48]. When turnover rates are chronically high, it may indicate that staff working in that hospital unit are not satisfied at work [46] or that the work environment is not favorable to psychological well-being [48]. Research on turnover rates indicates average annual turnover rates for nurses are between 14% and 27% in Canada [49]. This finding is supported by data worldwide that found the average turnover rate for nurses to be between 15% and 36% [50,51]. For this study, we will use job turnover rates as a guide to target the areas that are more or less successful with retention.

As a secondary indicator for retention, vacant positions will also be analyzed for nurses and physicians within the Network. Using a similar strategy, the job vacancy rate will be assessed as an indicator for retention [52]. The 2 indicators, the turnover and job vacancy rates, will be analyzed together to target areas within the Network that are doing well with retention and areas with retention challenges.

We will then target these areas to recruit participants for individual and group discussions as described in section “Qualitative Approach: Data Collection and Analysis.” For physicians, position turnover rates are less reported, and researchers generally use the intention to leave as a proxy for turnover rates [53]. Of the little data available, the average turnover rate for physicians is between 4% and 25% [54].

### Quantitative Approach: Data Collection and Analysis

Component 1 (quantitative secondary data analysis): to achieve specific objective 1, secondary data from the Network will be used to illustrate the proportions and the tendencies in physician and registered nurse vacancies by zone in the Network. It will also measure the extent of the current shortage and determine if there are significant differences between regions and sectors (eg, psychiatry and obstetrics) to identify those that are more successful than those that are less successful. The data from the past 5 years related to age and gender, the number of years of service in the Network at the moment of departure, family status, type of professional (specialist vs generalist), hospital unit or service area, and geographic area will be analyzed. Information on turnover rates for physicians and registered nurses for the past 5 years will be compiled and analyzed from the Regional Plan of Physician Workforce descriptive data collected by the Network’s Medical Services. To supplement this information, data from the Network’s Human Resources Department regarding medical and nursing staff who have left their positions will be used. From these data, a profile of professionals who have left the Network can be created to tailor retention strategies and improve their effectiveness.

Since we plan to use secondary data on job vacancies and turnover rates, we will be limited to the data already collected and may face an information gap. Some team members have used quantitative approaches in their research for several years. Faced with sometimes incomplete databases, the team may have to develop data collection tools to collect more quantitative data to supplement the secondary data provided by the Network. At this time, the approach considered is a web-based survey to target employees (nurses and doctors) working at the Network and people (nurses and doctors) who have left. If a survey is used, validated questions will be used to measure factors that impact job satisfaction and intention to leave the current workplace [55-57]. The survey design will be multiple choice questions with a 7-point Likert scale to maintain validation from previous studies.

The statistical analysis for specific objective 1 will be discussed first. Next, we will conduct bivariate and multivariate correlational analyses to assess the influence of the factors comprised in our conceptual framework over health professionals’ intention to stay. Considering that participants’ sociodemographic characteristics (ie, sectors, gender, age, education, and years of service) may influence the relationships between the variables under study (ie, factors comprise in our conceptual framework), analyses will be conducted to determine the magnitude of their effect [58].

### Qualitative Approach: Data Collection and Analysis

Component 2 (qualitative data): to achieve specific objective 2, qualitative data will be collected through semistructured interviews, focus groups, and a document review, including gray literature such as media reports.

#### Semistructured Interviews

The interview guides were developed to take into consideration the factors listed in the conceptual framework (Figure 3). Participants will be asked to share their views on topics such as job satisfaction, autonomy in decision-making, leadership, and language used at work. The first set of semistructured interviews (approximately 30) will be conducted with physicians and registered nurses who have left the Network in the past 5 years. The second set of semistructured interviews (approximately 30) will be conducted with these same 2 types of professionals the Network currently employs. In sum, the data will be used to understand whether the reasons for retention and departure are more related to the external environment or whether they result from the internal environment. The specific number of semistructured interviews and focus groups is subject to change, depending on data saturation, that is, when the ability to obtain additional new information has been attained, and further coding is no longer needed [59]. If necessary, we will adjust our recruitment efforts to ensure gender participation that represents diversity in the work environment, considering that the proportion of women is higher than men in the health sector. The interviews will be completed on the web or in person to respect certain COVID-19 prevention strategies and enhance a better understanding of the current retention realities across the province (ie, rural and urban regions). There are 2 exclusion criteria for our study: physicians and registered nurses who have left the Network for more than 5 years and those who have retired.

#### Focus Groups

The focus groups, which will also allow the achievement of specific objective 2, will be held with 5 to 10 participants in 4 different groups and with 2 distinct types of participants: leadership team and Board of Directors and Network managers. Participants will be asked about retention challenges in the workforce, strategies for retaining physicians and registered nurses, relationships with the Health Department and other stakeholders, and the involvement of OLMCs in retention efforts. The focus groups will be conducted at 3 different times (beginning, mid-term, and end of the study). The first focus group will be conducted at the very start of the project with key informants (leadership team, the executive team, and members of the governance team) to enhance the understanding pertaining to the factors responsible for past and current staff shortages. The last focus group, held at the end of our study with the same key informants, will be to mitigate and discuss retention strategies to enhance workplace satisfaction among nurses and physicians. In contrast, the 2 midpoint focus groups will target the Network’s management staff, more specifically: the first group in a service area where retention rates have been identified as low and the second group in a service area where retention rates have been identified as high. The information gathered in these latter 2 focus groups will be used to better understand the factors that influence physician and nurse retention in particular areas.

#### Document Review

A document review is another strategy that complements our data collection. Thus, documentary sources will allow us to corroborate the information obtained through our interviews and focus groups. We believe the document review will also be used to mark key retention milestones for the Network (province announcements, new internal regulations, procedures, etc). Different types of governance or administrative documents will be analyzed: Network and Department of Health annual reports, action plans, regulations, bills, etc. Since the Network does not have total control over the external and internal factors of its environment and since these can influence the retention of professionals, we consider it worthwhile to conduct a media review to understand their impact. The period for the document and media analysis is from March 2008, when the Network was created, to December 2022, when our data collection ended.

In terms of qualitative analysis, the data analysis approach used in the study is descriptive, where an initial analysis emerges from the respondents’ words, using a thematic approach to analysis [44,60]. Coding will be done iteratively in collaboration with other members of the research team using a predeveloped codebook. The team will code the same transcripts and then compare initial findings to help ensure intercoder reliability. Braun and Clark [60] approach to thematic analysis will be used to explore the current state of retention. The approach will also rely on a direct thematic analysis based on the “Magnet Hospital” model and “Making it Work” framework.

### Ethics Approval

The project was approved by both ethical research boards: the Vitalité Health Network (101574) and the Université de Moncton (2122-105). A detailed consent form will be explained orally and in writing to all participants. Informed consent will be obtained for all participants before taking part in the individual interview and focus groups. The importance of privacy and confidentiality protection for the research team will also be expressed to all participants, as one of our research partners is their current or past employer. Thus, each participant will be given a numerical code to preserve their anonymity. The code will be kept in a locked cabinet that only the 2 primary researchers can access. As for result reporting, such as in publication or recommendation reports, it will be done so the participants cannot be identified.

### Recruitment

Participants in the current workforce will be identified using a “snowball” technique [61]. Initial meetings with physicians and registered nurses associated with the Network should allow us to identify additional participants from these 2 disciplines who can inform us about the shortage of professional resources within the organization. A research team member will contact potential participants by phone or email to explain the research project and solicit their participation.

For respondents who have left the Network, we will seek the Network’s Human Resources support to identify potential candidates. Human Resources Department will then send an email to them, including the invitation to participate and specifying that the research is independent of the employer and that the latter will not have access to the data. The study details and the information on how to join the research team will be included in this invitation message. This procedure will ensure that no confidential employee information will be transferred to the research team. A CAD $20 (US $14.68) gift card will be offered to interview and focus group participants.

## Results

This study was funded in February 2022 by the Canadian Institutes of Health Research. Active enrollment and data collection started in the spring of 2022. A total of 56 semistructured interviews were conducted with physicians and nurses who are currently working for the network or have left in the past 5 years. The data collection for both quantitative and qualitative is intended to end by February 2023. Summer and fall 2023 is the anticipated period to disseminate the results locally, nationally, and internationally.

## Discussion

According to the World Health Organization, there is a global shortage of more than 7 million health professionals [62]. The uneven distribution of professional resources contributes to significant health disparities between rural and urban communities [62,63], and may be associated with economic stagnation and social decline in communities located in rural areas [64,65]. The province of New Brunswick is no exception to this situation, and the Network needs to effectively address its health workforce staff shortages as it may affect its viability.

Through the application of the “Making it work” and “Magnet Hospital” frameworks outside of urban settings, this study will contribute globally to the current reflection on the professional resources shortages within OLMCs [1]. The results of this study will help our partner (the Network) in identifying and implementing concrete strategies to promote physician and registered nurse retention by considering the particularities of its environment. It will allow the Network to replenish its strength and vitality by questioning issues such as autonomy [66], job satisfaction [40,67], and attachment to the community [42,68].

The results of this study will be published in a peer-reviewed open-access journal to increase public dissemination of the findings. The different knowledge dissemination activities will raise awareness among coresearchers and decision makers on the importance of addressing professional shortages in rural areas, especially within OLMCs.

The main limitation of this research is the availability of physicians and registered nurses due to pandemic fatigue and shortages in staffing. A second limitation to be considered in this study, pertaining to organizational systems and retention among physicians and nurses, is social desirability. Although confidentiality and anonymity will be respected and always enforced during the study, there is the potential and willingness to answer questions in a manner that will be viewed favorably by others (social desirability bias) [69]. A third limitation is related to the secondary data on job vacancies and turnover rates and the fact that we are constrained by the information that is already collected by the Network.

## Abbreviations

OLMC: Official Language Minority Community

## References

[1] Utzschneider A, Landy M (2018). Impacts of studying in a regional medical campus on practice location. Can Med Ed J.

[2] (2015). Plan des services cliniques, Phase 1 : Développement des services de santé primaires. Réseau de santé Vitalité.

[3] Beauchamp J, Bélanger M, Schofield A, Bordage R, Donovan D, Landry M (2013). Recruiting doctors from and for underserved groups: does new Brunswick's initiative to recruit doctors for its linguistic minority help rural communities?. Can J Public Health.

[4] Bélanger M, Bouchard L, Gaboury I, Sonier B, Gagnon-Arpin I, Schofield A, Bourque PE (2011). Perceived health status of Francophones and Anglophones in an officially bilingual Canadian province. Can J Public Health.

[5] Bouchard L, Gaboury I, Chomienne MH, Gilbert A, Dubois L (2009). Health in language minority situation. Health Policy.

[6] (2022). Data tables, 2021 census of population. Statistics Canada.

[7] Who are we. Vitalité Health Network.

[8] (2021). Pénurie de ressources humaines dans le système de santé : le Réseau fait le point sur l’état de la situation. Réseau de santé Vitalité.

[9] Delattre S (2021). Le recrutement, la priorité de Vitalité. Acadienouvelle.

[10] MacKinnon BJ (2022). Campbellton obstetric services won't reopen soon, says head of Vitalité. CBC News.

[11] Reni K, Lapointe S (2022). N.B. hospital loses fourth oncologist within weeks. Global News.

[12] (2021). Striving for dependable public health care: a discussion paper on the future of health care in New Brunswick. Government of New Brunswick.

[13] (2019). Making it Work: a framework for rural and remote workforce stability. Northern Periphery and Arctic Programme.

[14] Strasser R (2019). Remote rural workforce stability forum report. Making it Work.

[15] McClure ML, Poulin MA, Sovie MD, Wandelt MA (1983). Magnet hospitals. Attraction and retention of professional nurses. ANA Publ.

[16] Evans BC, Coon DW, Ume E (2011). Use of theoretical frameworks as a pragmatic guide for mixed methods studies: a methodological necessity?. J Mix Methods Res.

[17] Strasser R, Cheu H (2018). Needs of the many: Northern Ontario School of Medicine students' experience of generalism and rural practice. Can Fam Physician.

[18] Strasser R, Hogenbirk JC, Minore B, Marsh DC, Berry S, McCready WG, Graves L (2013). Transforming health professional education through social accountability: Canada's Northern Ontario School of Medicine. Med Teach.

[19] Strasser R (2018). Making it Work: a framework for remote rural workforce stability.

[20] (2019). Rapport sur les accomplissements 2018. École de médecine du Nord de l’Ontario.

[21] Attribution 4.0 International (CC BY 4.0). Creative Commons.

[22] Abelsen B, Strasser R, Heaney D, Berggren P, Sigurðsson S, Brandstorp H, Wakegijig J, Forsling N, Moody-Corbett P, Akearok GH, Mason A, Savage C, Nicoll P (2020). Plan, recruit, retain: a framework for local healthcare organizations to achieve a stable remote rural workforce. Hum Resour Health.

[23] McClure ML (2005). Magnet hospitals: insights and issues. Nurs Adm Q.

[24] Sibé M, Alis D (2016). L’hôpital magnétique : un hôpital "aimant" qui favorise performance et bien-être au travail. Stress, burn-out, harcèlement moral: De la souffrance au travail au management qualitatif.

[25] Grant B, Colello S, Riehle M, Dende D (2010). An evaluation of the nursing practice environment and successful change management using the new generation magnet model. J Nurs Manag.

[26] Luzinski C (2011). The Magnet model: an infrastructure for excellence. J Nurs Adm.

[27] Buffington A, Zwink J, Fink R, Devine D, Sanders C (2012). Factors affecting nurse retention at an academic Magnet® hospital. J Nurs Adm.

[28] Laschinger HKS, Almost J, Tuer-Hodes D (2003). Workplace empowerment and magnet hospital characteristics: making the link. J Nurs Adm.

[29] Drolet M, Bouchard P, Savard J, Gervais R (2017). Accessibilité et offre active. Santé et services sociaux en contexte linguistique minoritaire. Nouvelles perspectives en sciences sociales.

[30] Savard S, de Moissac D, Benoît J, Ba H, Zellama F, Giasson M, Drolet M, Drolet M, Bouchard P, Savard J (2017). Le recrutement et la rétention d'intervenants en santé et services sociaux bilingues en situation francophone minoritaire à Winnipeg et à Ottawa. Accessibilité et offre active: Soins de santé et services sociaux en contexte minoritaire.

[31] Art B, De Roo L, De Maeseneer J (2007). Towards unity for health utilising community-oriented primary care in education and practice. Educ Health (Abingdon).

[32] (2020). Les soins de santé n’ont jamais été aussi importants. Société Médicale du Nouveau-Brunswick.

[33] Bouchard P, Vézina S (2015). L’attraction organisationnelle et les valeurs des jeunes : le cas du personnel infirmier en milieu hospitalier. Min Ling Soc.

[34] de Moissac D, Drolet M, Savard S, Giasson F, Benoît J, Arcand I, Lagacé J, Dubouloz CJ, van Kemenade S, Drolet M, Savard J, Bouchard P (2017). Enjeux et défis dans l’offre de services dans la langue de la minorité: l’expérience des professionnels bilingues dans le réseau de la santé et des services sociaux. Accessibilité et offre active.

[35] Gagnon-Arpin I, Bouchard L, Leis A, Bélanger M (2014). Accès et utilisation des services de santé en langue minoritaire. La vie dans une langue officielle minoritaire au Canada.

[36] Bouchard P, Vézina S (2009). L'outillage des étudiants et des nouveaux professionnels: un levier essentiel pour l'amélioration des services de santé en français.

[37] Drolet M, Savard J, Benot J, Arcand I, Savard S, Lagacé J, Lauzon S, Dubouloz CJ (2014). Health services for linguistic minorities in a bilingual setting: challenges for bilingual professionals. Qual Health Res.

[38] Gauthier H (2011). Étude exploratoire sur les compétences linguistiques à l'embauche. Conseil communauté en santé du Manitoba.

[39] von Eiff A, von Eiff W, Ghanem M (2020). Magnet nursing—employee motivation, medical quality and patient satisfaction in unison. Health Manag.

[40] Brunelle Y (2009). Les hôpitaux magnétiques: un hôpital où il fait bon travailler en est un où il fait bon se faire soigner. Prat et Org des Soins.

[41] Sibé M (2020). Quelles transformations attendues? Quelles démarches inspirantes?. Actualité et dossier en santé publique.

[42] Collin S (2021). Lumière sur la réforme du système de santé au Nouveau-Brunswick: Évolution, jeux d’acteurs et instruments.

[43] Strasser RP (2017). Recruiting and retaining a rural medical workforce: the value of active community participation. Med J Aust.

[44] Creswell JW, Creswell JD (2018). Mixed methods procedures. Research Design: Qualitative, Quantitative, and Mixed Methods Approaches. 5th ed.

[44] Onwuegbuzie AJ, Teddlie C, Tashakkori A, Teddlie C (2003). A framework for analyzing data in mixed methods research. Handbook of Mixed Methods In Social and Behavioral Research.

[46] Buchan J (2010). Reviewing the benefits of health workforce stability. Hum Resour Health.

[47] Duffield CM, Roche MA, Homer C, Buchan J, Dimitrelis S (2014). A comparative review of nurse turnover rates and costs across countries. J Adv Nurs.

[48] Mathisen J, Nguyen TL, Jense JH, Rugulies R, Rod NH (2021). Reducing employee turnover in hospitals: estimating the effects of hypothetical improvements in the psychosocial work environment. Scand J Work Environ Health.

[49] O'Brien-Pallas L, Murphy GT, Shamian J, Li X, Hayes LJ (2010). Impact and determinants of nurse turnover: a pan-Canadian study. J Nurs Manag.

[50] Gess E, Manojlovich M, Warner S (2008). An evidence-based protocol for nurse retention. J Nurs Adm.

[51] Bae SH (2022). Noneconomic and economic impacts of nurse turnover in hospitals: a systematic review. Int Nurs Rev.

[52] Stoller JK, Sikon A, Schulte EE (2017). A perspective on crafting a dream medical career. OD Practitioner.

[53] (2021). Job vacancies, second quarter 2021. Statistics Canada.

[54] Willard-Grace R, Knox M, Huang B, Hammer H, Kivlahan C, Grumbach K (2019). Burnout and health care workforce turnover. Ann Fam Med.

[55] Kim H, Kim EG (2021). A meta-analysis on predictors of turnover intention of hospital nurses in South Korea (2000-2020). Nurs Open.

[56] Shen X, Jiang H, Xu H, Ye J, Lv C, Lu Z, Gan Y (2020). The global prevalence of turnover intention among general practitioners: a systematic review and meta-analysis. BMC Fam Pract.

[57] Park J, Min H (2020). Turnover intention in the hospitality industry: a meta-analysis. Int J Hosp Manag.

[58] Carlson KD, Wu J (2012). The illusion of statistical control. Organ Res Methods.

[59] Kuzel AJ, Crabtrree BF, Miller WL (1999). Sampling in qualitative inquiry. Doing Qualitative Research. 2nd ed.

[60] Braun V, Clarke V (2006). Using thematic analysis in psychology. Qual Res Psychol.

[61] Patton MQ (2002). Qualitative Research and Evaluation Methods.

[62] World Health Organization (2021). WHO Guideline on Health Workforce Development, Attraction, Recruitment and Retention in Rural and Remote Areas.

[63] Gillespie J, Cosgrave C, Malatzky C, Carden C (2022). Sense of place, place attachment, and belonging-in-place in empirical research: a scoping review for rural health workforce research. Health Place.

[64] Farmer J, Lauder W, Richards H, Sharkey S (2003). Dr. John has gone: assessing health professionals' contribution to remote rural community sustainability in the UK. Soc Sci Med.

[65] Prior M, Farmer J, Godden DJ, Taylor J (2010). More than health: the added value of health services in remote Scotland and Australia. Health Place.

[66] Junttila K, Heikkilä A, Heikkilä A, Koivunen M, Lehtikunnas T, Mattila E, Meriläinen M, Peltokoski J, Sneck S, Tervo-Heikkinen T (2023). The impact of leadership in the autonomy and engagement of nurses: a cross-sectional multicenter study among nurses outside the United States. J Nurs Adm.

[67] Kelly LA, McHugh MD, Aiken LH (2011). Nurse outcomes in Magnet® and non-magnet hospitals. J Nurs Adm.

[68] Beccaria L, McIlveen P, Fein EC, Kelly T, McGregor R, Rezwanul R (2021). Importance of attachment to place in growing a sustainable Australian rural health workforce: a rapid review. Aust J Rural Health.

[69] Bergen N, Labonté R (2020). "Everything is perfect, and we have no problems": detecting and limiting social desirability bias in qualitative research. Qual Health Res.

